# Diet-related inflammation is positively associated with atherogenic indices

**DOI:** 10.1038/s41598-024-63153-1

**Published:** 2024-06-08

**Authors:** Neda Heidarzadeh-Esfahani, Salimeh Hajahmadi, Yahya Pasdar, Mitra Darbandi, Farid Najafi, Mehdi Moradinazar, Mitra Bonyani, Roxana Feyz-BashiPoor, Shahin Soltani

**Affiliations:** 1 Research Center for Environmental Determinants of Health (RCEDH), Health Institute, Kermanshah University of Medical Sciences, Kermanshah, Iran; 2 Nutritional Sciences Department, School of Nutrition Sciences and Food Technology, Kermanshah University of Medical Sciences, Kermanshah, Iran; 3 Ala Cancer Control and Prevention Centre, Isfahan, Iran; 4 Department of Nutrition, School of Public Health, Shahid Sadoughi University of Medical Sciences, Yazd, Iran; 5 Medical Education Development Centre, Kermanshah University of Medical Sciences, Kermanshah, Iran; 6 Student Research Committee, Kermanshah University Of Medical Sciences, Kermanshah, Iran

**Keywords:** Dyslipidemia, Nontraditional lipid parameters, Lipoprotein ratios, Atherogenic risk, Dietary inflammatory index, Persian cohort, Cardiology, Diseases, Medical research

## Abstract

Current evidence suggests that non-traditional serum lipid ratios are more effective than traditional serum lipid parameters in predicting vascular diseases, and both of them are associated with dietary patterns. Therefore, this study aimed to investigate the relationship between the dietary inflammatory index (DII) and atherogenic indices using traditional serum lipid parameters (triglyceride (TG), total cholesterol (TC), LDL cholesterol (LDL–c), high-density lipoprotein cholesterol (HDL–c)) and non-traditional serum lipid ratios (atherogenic index of plasma (AIP), Castelli's index-I (CRI_I), Castelli's index-II (CRI_II), the lipoprotein combination index (LCI), and the atherogenic coefficient (AC)). Basic information from the Ravansar Non-Communicable Diseases cohort study was utilized in the present cross-sectional observational study. The study included 8870 adults aged 35–65 years. A validated food frequency questionnaire (FFQ) was used to measure DII. We compared the distributions of outcomes by DII score groups using multivariable linear regression. The difference between DII score groups was evaluated by the Bonferroni test. The mean ± SD DII was − 2.5 ± 1.43, and the prevalence of dyslipidemia was 44%. After adjusting for age, sex, smoking status, alcohol consumption status, physical activity, systolic blood pressure (SBP), diastolic blood pressure (DBP), fasting blood sugar (FBS), body mass index (BMI) and socioeconomic status (SES), participants in the highest quartile of DII had a greater risk for CRI_I (β = 0.11, CI 0.05, 0.18), CRI_II (β = 0.06, CI 0.01, 0.11), LCI (β = 0.11, CI 288.12, 8373.11), AC (β = 0.11, CI 0.05, 0.17) and AIP (β = 0.06, CI 0.02, 0.10). Moreover, according to the adjusted logistic regression model, the risk of dyslipidemia significantly increased by 24% (OR: 1.24, 95% CI 1.08–1.41), 7% (OR: 1.07, 95% CI 0.94, 1.21) and 3% (OR: 1.03, 95% CI 0.91, 1.16) in Q4, Q3 and Q2 of the DII, respectively. Finally, diet-related inflammation, as estimated by the DII, is associated with a higher risk of CRI-I, CRI-II, LCI, AC, and AIP and increased odds of dyslipidemia.

## Introduction

Dyslipidemia, characterized by an imbalance in lipid profiles, is a recognized factor in the development of atherosclerosis and cardiovascular diseases (CVDs)^1^. The World Health Organization (WHO) estimates that CVDs are a major cause of mortality, with 7.9 million deaths annually^2^. Furthermore, the global prevalence of lipid profile abnormalities is increasing worldwide^3^. Elevated levels of low-density lipoprotein cholesterol (LDL-C) and a decrease in high-density lipoprotein (HDL-C) cholesterol result in the accumulation of plaque throughout the arteries and increase atherosclerotic CVD risk^4^. Alongside these prime parameters, lipoprotein ratios such as total-C/HDL-C and LDL-C/HDL-C and the logarithm of the triglyceride (TG)/HDL-C ratio have greater predictive capacity for CVD incidence and the severity of atherosclerosis^5,6^. Atherosclerosis is attributed to several inflammatory cells and factors throughout the initiation, progression and formation of atherosclerotic plaques^4,7^.

Previous evidence indicates that the intake of various dietary agents can lead to the modulation and attenuation of systemic inflammation^8–11^. Indeed, certain dietary patterns, such as Mediterranean and prudent diets or high loads of dietary intake of fruits, vegetables, whole grains, polyunsaturated fatty acids, fiber, and nonnutritive compounds (e.g., polyphenols), were inversely associated with biomarkers of inflammation and endothelial activation, such as interleukin-18 (IL-18), fibrinogen, C-reactive protein (CRP), interleukin-6 (IL-6), and cell adhesion molecules, which can lead to CVD and atherosclerosis^12–14^. In contrast, Western dietary patterns with high loadings of saturated fatty acids, red meat, processed meat, dessert, beer, sugar-sweetened beverages and low-fiber foods were positively related to inflammatory biomarkers^12^. The Dietary Inflammatory Index (DII) was designed as an applicable diet-related, literature-based tool in human populations^15^. The DII evaluates the overall inflammatory potential of dietary components, such as macronutrients, micronutrients, phytochemicals, and other dietary components, based on pro- and anti-inflammatory aspects^16^.

In recent years, there have been several Umbrella reviews of systematic reviews and meta-analyses of observational studies of links between noncommunicable diseases and all causes of mortality with DII. Higher DII scores were related to harmful effects on health outcomes^16–19^. However, few studies have investigated the association between DII and lipoprotein ratios in developing Middle Eastern countries^20,21^. Frequent studies have shown that lipoprotein ratios are a greater predictor of cardiovascular risk than lipid profiles are in developing countries^22–24^. Moreover, lipoprotein ratios better reflect metabolic and clinical interactions with lipid fractions. To reduce the economic and clinical burden of dyslipidemia, dietary modifications may be a crucial strategy. The DII can be used to evaluate overall inflammation related to nutritional patterns. This study aimed to examine whether the DII score is related to dyslipidemia and atherogenic risk.

## Methods and materials

### Data source and study population

**Figure 1:**
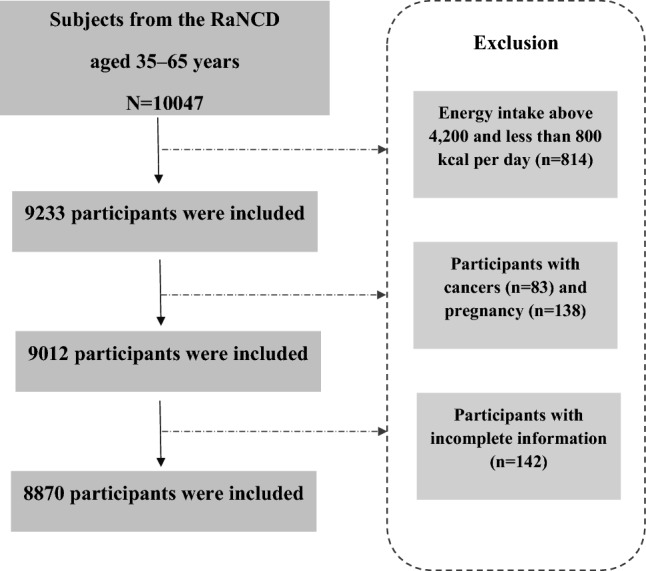
Flow chart of the study subjects.

We carried out an analysis using data from 10,047 adults aged 35–65 years who participated in the Ravansar Noncommunicable Diseases Cohort Study (RaNCD) as a part of the Prospective Epidemiological Research Studies in Iran (PERSIAN). The present cross-sectional study included 4030 men and 4840 women and was started in November 2014 until February 2017. The study design, objectives, and procedures of the RaNCD study have been published elsewhere before^25–27^. In this observational study, data were retrieved from adults who completed the dietary interview, and the exclusion criteria were as follows: individuals with energy intake above 4200 and less than 800 kcal per day (n = 814), cancer (n = 83), pregnancy (n = 138), and incomplete information (n = 142). Overall, 8870 participants were included in the study (Fig. 1).

### Dietary data and dietary inflammatory index calculations

In total, 118 food items were assessed by the validated food frequency questionnaire based on portion size and daily, weekly, monthly, or annual food consumption through face-to-face interviews. The questionnaire was previously validated in the Iranian population^28^. We assessed the inflammatory potential of the whole diet by DII scores according to the recent method proposed by Shivappa et al.^15^. The DII is a score-based algorithm that scores 45 dietary constituents in the range of –1 (maximally anti-inflammatory) to + 1 (maximally proinflammatory). The DII evaluates the inflammatory status of the whole diet by predicting the levels of specific inflammatory and anti-inflammatory markers, including C-reactive protein (CRP), interleukin-1 (IL-1), interleukin-4 (IL-4), interleukin-6 (IL-6), interleukin-8 (IL-8), interleukin-10 (IL-10), and tumor necrosis factor α (TNF-α). Similarly, in this study, the DII was calculated using a total of 31 available food parameters from the FFQ of the 45 possible food variables composing the DII: retinol, beta-carotene, pyridoxine, cobalamin, ascorbic acid, calciferol, tocopherol, folic acid, niacin, thiamine, riboflavin, iron, zinc, selenium, magnesium, omega 3, omega 6, total fat, saturated fats (SFAs), cholesterol, monounsaturated fatty acids (MUFAs), polyunsaturated fatty acids (PUFAs), fiber, protein, total fat, carbohydrate and energy, caffeine, onion, garlic, and tea.

### Lipid profile measurements

The traditional and nontraditional lipid profile parameters used for atherogenic indices included triglyceride (TG), total cholesterol (TC), LDL cholesterol (LDL–c), high-density lipoprotein cholesterol (HDL–c), the atherogenic index of plasma (AIP), Castelli's index-I (CRI_I), Castelli's index-II (CRI_II), the lipoprotein combination index (LCI), and the atherogenic coefficient (AC).

Lipid profile measurements have been previously described in detail^27^. Lipoprotein ratio measurements were calculated as AIP: log (TG/HDL–C)^29^, AC: ((TC- HDL–C)/HDL–C)^30^, CRI-I: (TC/HDL–C)^31^, CRI-II: (LDL–C/HDL–C) and LCI: (TC*TG*LDL-C)/HDL-C^31^. Dyslipidemia was defined as LDL-C ≥ 160 mg/dL and/or TC ≥ 240 mg/dL and/or HDL-C < 40 mg/dL and/or TG > 200 mg/dL^32^.

### Anthropometric and physical activity data

Weight, height, body mass index (BMI), waist-to-hip ratio (WHR), visceral fatty acid (VFA), and percentage of body fat (PBF) were collected and analyzed in this study. The anthropometric data were measured using an automated bioelectric impedance machine (In Body 770, BIOSPACE KOREA). Physical activity levels were evaluated according to the PERSIAN cohort self-reported questionnaire, and participants' responses were measured in terms of the metabolic equivalent of task per hour per day (MET/h per day), following a methodology from a separate study^25^. Physical activity was categorized into three levels: low (24–36.5 MET/hour per day), moderate (36.6–44.4 MET/hour per day) and high (≥ 44.5 MET/hour per day)^25,33^.

### Other variables

In the present study, the sociodemographic information included sex, age, region (rural or urban), socioeconomic status (poor, middle, or rich) and alcohol consumption (yes or no). Patients were divided into four groups according to their self-reported smoking status: current smokers, former smokers, passive smokers, and nonsmokers. Blood pressure and fasting blood glucose levels were measured.

### Statistical analysis

All the statistical analyses were performed using the software STATA version 14.2 (Stata Corp., College Station, TX, USA). ANOVA and chi-square tests were used to determine the significance of differences between continuous variables and categorical variables across quartiles of DII scores. To compare the differences in the continuous variables among four DII score groups, Bonferroni (post-hoc test) was used after one-way analysis of variance, while categorical variables were compared using the χ2 test. The associations between DII scores and dyslipidemia were determined via logistic regression models, while the relationships between DII scores and atherogenic risk scores were determined via linear regression models. P values < 0.05 and 95% confidence intervals (CIs) were considered to indicate statistical significance.

### Ethics approval and consent to participate

The study was approved by the ethics committee of Kermanshah University of Medical Sciences (IR.KUMS.REC.1401.560). All methods were carried out in accordance with relevant guidelines and regulations. All the participants provided oral and written informed consent.

## Results

The demographic and socioeconomic characteristics of our participants are shown in Table 1. The mean age of the participants at baseline was 47.49 ± 8.23 years. Fifty-four percent of the participants were female. The prevalence of dyslipidemia was 44%. The means ± SDs of LDL-c, HDL-c, TC and TG were 111.63 ± 31.33 (mg/dl), 46.60 ± 11.37 (mg/dl), 185.59 ± 37.73 (mg/dl) and 136.82 ± 81.64 (mg/dl), respectively. The mean ± SD DII was − 2.5 ± 1.43. The participants with dyslipidemia had greater pro-inflammatory scores (-2.42 ± 1.46) than those with a normal lipid profile (-2.57 ± 1.41) (*P* < 0.001). Participants with dyslipidemia reported having a greater BMI, WHR, VFA, SBP and DBP (*P* < 0.001). Moreover, those in the dyslipidemia category were more likely to have higher CRI_I (4.89 ± 1.06 vs. 3.58 ± 0.73) and CRI_II (2.92 ± 0.84 vs. 2.17 ± 0.62) scores than were those in the non-dyslipidemia category (*P* < 0.001), as were those in the LCI (108,769.8 ± 84,434.68 vs. 44,135.68 ± 28,228.21), AIP (1.41 ± 0.56 vs. 0.62 ± 0.46) and AC (3.88 ± 1.06 vs. 0.62 ± 0.46).

**Table 1:** Characteristics of participants according to the presence or absence of dyslipidaemia.

Characteristics	Total (n = 8870)	Non- dyslipidaemia (n = 4989)	Dyslipidaemia (n = 3881)	P-value*
	Mean ± SD or frequency (%)			
Age (year)	47.49 ± 8.23	46.96 ± 8.21	48.18 ± 8.32	< 0.001
Gender				
Male	4030 (45.4%)	1902 (38.12%)	2128 (54.83%)	< 0.001
Female	4840 (54.6%)	3087 (61.88%)	1753 (45.17%)	
Residency, n (%)				
Urban	5227	2765 (55.42%)	2462 (63.44%)	< 0.001
Rural	3643	2224 (44.58%)	1419 (36.56%)	
Socio-economic status				
Poor	2977	1759 (35.26%)	1218 (31.41%)	< 0.001
Middle	2943	1647 (33.01%)	1296 (33.42%)	
Rich	2947	1583 (31.73%)	1364 (33.24%)	
Physical activity (Met-h/day)				
Light	2728	1386 (27.78%)	1342 (34.58%)	< 0.001
Moderate	4280	2516 (50.43%)	1764 (45.45%)	
High	1862	1087 (21.79%)	775 (19.97%)	
Smoking status				
Current smoker	966	420 (8.46%)	546 (14.13%)	< 0.001
Former smoker	739	357(7.19%)	382 (9.89%)	
Passive smoker	3427	1947 (39.22%)	1480 (38.31%)	
No smoker	3695	2240 (45.12%)	1455 (37.67%)	
Alcohol drinking				
Yes	8490	165 (3.31%)	215 (5.54%)	< 0.001
No	380	4824 (96.69%)	3666 (94.46%)	
BMI (kg/m^2^)	27.46 ± 4.63	26.98 ± 4.83	28.07 ± 4.27	< 0.001
WHR	0.94 ± 0.06	0.93 ± 0.06	0.95 ± 0.05	< 0.001
PBF (%)	34.12 ± 9.43	34.17 ± 9.88	34.06 ± 8.81	0.583
VFA (kg)	122.87 ± 51.49	120.72 ± 53.87	125.63 ± 48.11	< 0.001
SBP (mmHg)	108.10 ± 17.01	106.32 ± 16.57	110.39 ± 17.30	< 0.001
DBP (mmHg)	69.73 ± 9.90	68.80 ± 9.64	70.93 ± 10.09	< 0.001
FBS (mg/dl)	97.09 ± 29.65	93.94 ± 25.29	101.13 ± 34.03	< 0.001
LDL (mg/dl)	111.63 ± 31.33	109.21 ± 25.05	114.74 ± 37.68	< 0.001
HDL (mg/dl)	46.60 ± 11.37	51.78 ± 9.37	39.94 ± 10.18	< 0.001
TC (mg/dl)	185.59 ± 37.73	181.42 ± 28.29	190.94 ± 46.64	< 0.001
TG (mg/dl)	136.82 ± 81.64	02.26 ± 37.88	181.26 ± 99.40	< 0.001
CRI-I	4.15 ± 1.10	3.58 ± 0.73	4.89 ± 1.06	< 0.001
CRI_II	2.50 ± 0.81	2.17 ± 0.62	2.92 ± 0.84	< 0.001
LCI	72,422.2 ± 67,793.16	44,135.68 ± 28,228.21	108,769.8 ± 84,434.68	< 0.001
AC	3.15 ± 1.10	2.58 ± 0.73	3.88 ± 1.06	< 0.001
AIP	0.96 ± 0.64	0.62 ± 0.46	1.41 ± 0.56	< 0.001
DII	− 2.5 ± 1.43	− 2.57 ± 1.41	− 2.42 ± 1.46	< 0.001

*BMI* body mass index, *WHR* waist-to-hip ratio, PBF percentage of body fat, *VFA* visceral fatty acid, *SBP* systolic blood pressure, *DBP* diastolic blood pressure, *FBS* fasting blood sugar, *LDL* low-density lipoprotein, *HDL* high-density lipoprotein, *TC* total cholesterol, *TG* triglyceride, *CRI-I* Castelli's index-I, *CRI-II* Castelli's index-II, *LCI* lipoprotein combination index, *AC* atherogenic coefficient, *AIP* atherogenic index of plasma, *DII* dietary inflammatory index.

Chi-square test or T-test were used.

*P-value < 0.05.

As shown in Table 2, in the highest quartile of the DPI, the intake of saturated fat, dairy products, calcium, and selenium was lower than that in the lowest quartile (*P* < 0.001). Conversely, individuals in the higher DII quartile had significantly greater intakes of energy, protein, MUFAs, PUFAs, whole grains, fruits, vegetables, red and white meat, legumes, eggs, fibers, nuts, vitamin E, vitamin A, vitamin D, vitamin K, vitamin C, vitamin B6, vitamin B12, magnesium, zinc, and iron (*P* < 0.001). Additionally, there were no statistically significant differences in carbohydrate or fat consumption across the DII quartiles. Moreover, the difference between DII score groups was evaluated by the Bonferroni test.

**Table 2:** Table showing Mean, Standard Deviation and Standard Error of dietary intakes of participants across quartiles of DII.

Dietary intake	Dietary inflammatory index scores				P-value
	Q1	Q2	Q3	Q4	
Frequency	2366	2349	2298	1857	–
Min, Max	− 6.18, − 3.54	− 3.54, − 2.68	− 2.68, − 1.38	− 1.38, 3.83	–
Energy(kcal/d)**	2162.43 ± 665.6^abc^	2370.3 ± 653.47^de^	2666.96 ± 687.78^f^	3024.58 ± 662.9367	< 0.001
Carbohydrate (%EI)**	61.43 ± 6.59	61.50 ± 6.17	61.44 ± 6.00	61.45 ± 5.96	0.979
Protein (%EI)**	13.15 ± 1.99^abc^	13.52 ± 2.07^de^	13.85 ± 2.11^f^	14.60 ± 2.35	< 0.001
Fat (%EI)**	26.59 ± 6.34	26.74 ± 6.00	26.94 ± 5.73	26.87 ± 5.62	0.201
Saturated fat (g/d)*	29.87 ± 0.19^abc^	28.55 ± 0.19^de^	27.89 ± 0.19^f^	26.30 ± 0.22	< 0.001
MUFA (g/d)*	18.63 ± 0.14^abc^	19.29 ± 0.14^de^	19.90 ± .14^f^	20.45 ± 00.16	< 0.001
PUFA (g/d)*	9.37 ± 0.08^abc^	10.63 ± 0.08^de^	11.74 ± 0.08^f^	13.04 ± 0.10	< 0.001
Whole grains (g/d)*	6.51 ± 0.25^abc^	8.4 ± 0.25^de^	10.82 ± 0.25^f^	15.5 ± 0.3	< 0.001
Dairy (g/d)*	501.4 ± 7.32^abc^	441.16 ± 7.12^de^	429.4 ± 7.2^f^	380.55 ± 8.4	< 0.001
Fruits (g/d)*	189.41 ± 3.81^abc^	237.63 ± 3.71^de^	300.27 ± 3.74^f^	380.97 ± 4.37	< 0.001
Vegetables (g/d)*	298.97 ± 4.04^abc^	397.85 ± 3.93^de^	507.63 ± 3.97^f^	735.75 ± 4.64	< 0.001
Red and white meat (g/d)*	70.26 ± 1.04^abc^	73.04 ± 1.01^de^	77.76 ± 1.02^f^	86.55 ± 1.2	< 0.001
Legumes (g/d)*	21.22 ± 0.56^abc^	27.81 ± 0.54^de^	35.07 ± 0.54^f^	52.41 ± 0.64	< 0.001
Egg (g/d)*	18.03 ± 0.038^abc^	20.60 ± 0.37^de^	20.72 ± 0.38^f^	21.60 ± 0.44	< 0.001
Fibre (g/d)*	19.97 ± 0.10^abc^	22.74 ± 0.10^de^	26.16 ± 0.10^f^	32.2 ± 0.12	< 0.001
Nuts(g/d)*	6.17 ± 0.20^abc^	7.47 ± 0.20^de^	9.33 ± 0.20^f^	10.91 ± 0.23	< 0.001
Vitamin E (mg/d)*	6.01 ± 0.56^abc^	6.96 ± 0.05^de^	8.03 ± 0.05^f^	9.56 ± 0.06	< 0.001
Vitamin A (mg/d*	5009.03 ± 81.81^abc^	6541.27 ± 79.58^de^	9067 ± 80.31^f^	13,844.2 ± 93.84	< 0.001
Vitamin D (IU/d)*	33.71 ± 0.60^abc^	39.92 ± 0.58^de^	45.54 ± 0.59^f^	53.69 ± 0.69	< 0.001
Vitamin K (mg/d)*	104.63 ± 2.4^abc^	140.75 ± 2.33^de^	193.12 ± 2.35^f^	312.68 ± 2.75	< 0.001
Vitamin C (mg/d)*	72.52 ± 1.03^abc^	92.58 ± 1.00^de^	116.7 ± 1.01^f^	164.30 ± 1.18	< 0.001
Vitamin B6 (mg/d)*	7.59 ± 0.17^abc^	9.71 ± 0.17^de^	11.77 ± 0.17^f^	15.33 ± 0.20	< 0.001
Vitamin B12 (mg/d)*	5.53 ± 0.09^abc^	6.15 ± 0.09^de^	7.20 ± 0.09^f^	8.80 ± 0.11	< 0.001
Calcium (mg/d)*	1293.45 ± 5.62^abc^	1241.99 ± 5.47^de^	1220.01 ± 5.52^f^	1194.26 ± 6.45	< 0.001
Magnesium (mg/d)*	288.16 ± 0.95^abc^	315.50 ± 0.93^de^	343.43 ± 0.93^f^	399.98 ± 1.09	< 0.001
Selenium (mg/d)*	120.85 ± 0.58^abc^	118.91 ± 0.57^de^	116.93 ± 0.57^f^	117.02 ± 0.67	< 0.001
Zinc (mg/d)*	9.30 ± 0.04^abc^	9.62 ± 0.03^de^	10.13 ± 0.035^f^	11.00 ± 0.04	< 0.001
Iron (mg/d)*	17.78 ± 0.07^abc^	17.98 ± 0.07^de^	18.28 ± 0.07^f^	19.63 ± 0.08	< 0.001

*P-value < 0.05, P value obtained by Bonferroni (post-hoc test) after one-way analysis of variance.

^a^There is a statistically significant difference between the average in DII scores 1 and 2.

^b^There is a statistically significant difference between the average in DII scores 1 and 3.

^c^There is a statistically significant difference between the average in DII scores 1 and 4.

^d^There is a statistically significant difference between the average in DII scores 2 and 3.

^e^There is a statistically significant difference between the average in DII scores 2 and 4.

^f^There is a statistically significant difference between the average in DII scores 3 and 4.

SE* standard error, SD** standard deviation.

The lipid profile and atherogenic indices of the participants across DII quartiles are shown in Table 3 and the difference between DII score groups was evaluated by Bonferroni test.

**Table 3:** Mean and confidence interval of lipid profile and atherogenic indexes of participants across quartiles of dietary inflammatory index scores.

Dietary intake	Dietary inflammatory index scores				P-value*
	Q1	Q2	Q3	Q4	
LDL (mg/dl)**	112.40 ± 32.20	112.24 ± 31.11	111.14 ± 30.29	110.48 ± 31.74	0.146
HDL (mg/dl)**	48.07 ± 11.58^abc^	47.04 ± 11.32^de^	46.01 ± 11.35^f^	44.89 ± 10.89	< 0.001
TC (mg/dl)**	187.35 ± 38.78^c^	186.02 ± 37.21	184.51 ± 36.73	184.11 ± 38.18	0.017
TG (mg/dl)**	134.41 ± 80.91	133.68 ± 75.17	136.87 ± 79.48	143.81 ± 92.04^cef^	< 0.001
CRI_I**	4.06 ± 1.09^bc^	4.12 ± 1.08	4.18 ± 1.08	4.28 ± 1.15^ef^	< 0.001
CRI_II**	2.44 ± 0.81^bc^	2.49 ± 0.81^e^	2.52 ± 0.79	2.56 ± 0.83	< 0.001
LCI**	70,927.26 ± 68,437.11	70,224.04 ± 62,819.01	72,051.39 ± 65,125.54	77,568.9 ± 75,611.4^ce^	0.002
AC**	3.06 ± 1.09^bc^	3.12 ± 1.08	3.18 ± 1.08	3.28 ± 1.15^ef^	< 0.001
AIP**	0.91 ± 0.65^bc^	0.94 ± 0.62	0.98 ± 0.63	1.04 ± 0.65^ef^	< 0.001

*P-value < 0.05, P value obtained by Bonferroni (post-hoc test) after one-way analysis of variance.

^a^There is a statistically significant difference between the average in DII scores 1 and 2.

^b^There is a statistically significant difference between the average in DII scores 1 and 3.

^c^There is a statistically significant difference between the average in DII scores 1 and 4.

^d^There is a statistically significant difference between the average in DII scores 2 and 3.

^e^There is a statistically significant difference between the average in DII scores 2 and 4.

^f^There is a statistically significant difference between the average in DII scores 3 and 4.

S.D** standard deviation, *TG* triglyceride, *LDL* low-density lipoprotein cholesterol, *HDL* high-density lipoprotein cholesterol, *TC* total cholesterol, *AC* Atherogenic coefficient, *CRI* Castelli Risk Index.

A higher DII was related to higher TG, CRI_I, CRI_II, LCI, AC and AIP (*P* < 0.001) in patients in the highest DII quartiles than in those in the lowest DII quartile. Participants in the upper quartile had lower levels of high-density lipoprotein cholesterol (HDL-C) and total cholesterol (TC) than those in the lower quartile.

There was a direct association between DII and the risk of prevalent dyslipidemia according to the crude odds ratios (ORs) (Table 4).

**Table 4:** Association between the dietary inflammatory index scores and dyslipidemia by logistic regression models.

	Dietary inflammatory index scores				P-trend
	Q1	Q2 OR (95% CI)	Q3 OR (95% CI)	Q4 OR (95% CI)	
Model 1	1	1.04 (0.93, 1.017)	1.12 (0.99, 1.26)	1.36 (1.20, 1.54)	< 0.001
Model 2	1	1.04 (0.92, 1.17)	1.11 (0.98, 1.25)	1.32 (1.16, 1.50)	< 0.001
Model 3	1	1.03 (0.91, 1.16)	1.07 (0.94, 1.21)	1.24 (1.08, 1.41)	0.001

*OR* Odds ratio, *CI* Confidence interval.

Model 1: Unadjusted model.

Model 2: Adjusted for age and sex.

Model 3: Adjusted for age, sex, smoking status, alcohol drinking, physical activity, SBP, DBP, FBS, BMI and SES.

After adjustment for age and sex, a higher DII was associated with a greater likelihood of dyslipidemia. The corresponding odds ratios were 1.32 (95% CI 1.16, 1.50), 1.11 (95% CI 0.098, 1.25) and 1.04 (95% CI (0.92, 1.17)) for the fourth, third and second quintiles of the DII, respectively, compared with the first. According to the fully adjusted logistic regression models and after adjustment for participant age, sex, smoking status, alcohol consumption status, physical activity, SBP, DBP, FBS, BMI and SES, the risk of dyslipidemia significantly increased by 24% (OR: 1.24, 95% CI 1.08–1.41), 7% (OR: 1.07, 95% CI 0.94, 1.21) and 3% (OR: 1.03, 95% CI 0.91, 1.16) in quartile 4, quartile 3 and quartile 2 of the DII, respectively, compared with the first quartile.

We also analyzed the linear relationship between DII and atherogenic indices (Table 5). The linear regression model showed that the DII was positively associated with atherogenic indices (CRI_I, CRI_II, LCI, AC and AIP). Indeed, in the highest DII quartile, atherogenic indices were significantly greater than those in the lowest quartile according to both unadjusted and adjusted models.

**Table 5:** Association between the dietary inflammatory index scores and and atherogenic indexes by linear regression models.

Atherogenic indexes		Dietary inflammatory index scores			
		Q1	Q2	Q3	Q4
CRI_I**	Model 1 β (95% CI)	1	0.06 (− 0.03, 0.12)	0.11 (0.05, 0.17)	0.21 (0.15, 0.28)
	Model 2 β (95% CI)	1	0.05 (0.00, 0.11)	0.10 (0.04, 0.16)	0.18 (0.12, 0.25)
	Model 3 β (95% CI)	1	0.03 (− 0.02, 0.09)	0.06 (0.00, 0.12)	0.11 (0.05, 0.18)
CRI_II**	Model 1 β (95% CI)	1	0.05 (0.00, 0.09)	0.07 (0.03, 0.12)	0.12 (0.07, 0.17)
	Model 2 β (95% CI)	1	0.05 (0.00, 0.09)	0.07 (0.03, 0.12)	0.11 (0.06, 0.16)
	Model 3 β (95% CI)	1	0.04 (0.00, 0.08)	0.05 (0.01, 0.09)	0.06 (0.01, 0.11)
LCI**	Model 1 β (95% CI)	1	− 703.22 (− 4571.39, 3164.95)	1124.13 (− 2765.95, 5014.21)	6641.64 (2523.73, 10,759.55)
	Model 2 β (95% CI)	1	− 304.98 (− 4144.05, 3534.08)	1724.86 (− 2146.47, 5596.21)	6904.86 (2796.47,11,013.24)
	Model 3 β (95% CI)	1	− 568.57 (− 4312.98, 3175.83)	739.44 (− 3065.90, 4544.8)	4330.61 (288.12, 8373.11)
AC**	Model 1 β (95% CI)	1	0.06 (0.00, 0.12)	0.11 (0.05, 0.17)	0.21 (0.14, 0.28)
	Model 2 β (95% CI)	1	0.05 (0.00, 0.11)	0.1 (0.04, 0.16)	0.18 (0.11, 0.25)
	Model 3 β (95% CI)	1	0.03 (− 0.02, 0.09)	0.06 (0.00, 0.12)	0.11 (0.05, 0.17)
AIP**	Model 1 β (95% CI)	1	0.03 (0.00, 0.06)	0.07 (0.03, 0.11)	0.13 (0.09, 0.17)
	Model 2 β (95% CI)	1	0.02 (0.00, 0.06)	0.06 (0.02, 0.1)	0.11 (0.07, 0.15)
	Model 3 β (95% CI)	1	0.00 (− 0.02, 0.04)	0.03 (0.00, 0.06)	0.06 (0.02, 0.10)

Model 1: Unadjusted model.

Model 2: Adjusted for age and sex.

Model 3: Adjusted for age, sex, smoking status, alcohol drinking, physical activity, SBP, DBP, FBS, BMI and SES.

A DII in the fourth quartile compared with the first quartile was positively associated with a higher risk of CRI_I (β = 0.11, CI 0.05, 0.18), CRI_II (β = 0.06, CI 0.01, 0.11), LCI (β = 0.11, CI 288.12, 8373.11), AC (β = 0.11, CI 0.05, 0.17) and AIP (β = 0.06, CI 0.02, 0.10) according to the fully adjusted linear regression model.

## Discussion

The results revealed that there was direct bonding between the DII score and dyslipidemia risk (based on crude ORs), and dyslipidemia risk (after adjustment for potentially confounding factors) significantly increased by 3%, 7% and 24% in Q2, Q3 and Q4 of the DII, respectively, compared with the first quartile. In addition, the DII was positively associated with atherogenic indices. A DII in the fourth quartile was positively associated with a higher risk of CRI_I, CRI_II, LCI, AC and AIP than was a DII in the first quartile.

Investigations of adults with metabolic syndrome^34,35^ or atherosclerosis^21^, children^36,37^, elderly individuals^38,39^ and overweight or obese women^40,41^ have also shown that following a diet with a high DII may significantly increase dyslipidemia, which is in line with our findings. A survey of the elderly population in Taiwan reported that the inflammatory dietary pattern in the male group was linked to a 20% increase in dyslipidemia risk, while in the female group, it showed a borderline rising trend in the index studied (OR: 1.12, 95% CI 0.99–1.26, p = 0.052)^42^.

Furthermore, a survey of Mexican adults demonstrated that an inflammatory diet could considerably increase hypertriglyceridemia risk (HRQ4 vs Q1 = 2.28; 95% CI 1.13, 4.57; P-trend = 0.01)^43^.

A cohort study of elderly individuals (55 years and older) over 5 years also demonstrated that there was a direct relationship between a diet with a higher DII and increasing plasma triglyceride levels, especially in people with abdominal obesity (per score increment: 1.62%, 95% CI 0.58–2.76%; pFDR = 0.01)^44^.

The bulk of evidence in the literature indicates that habitual intake of an inflammatory diet is directly related to unfavorable lipid profiles as well as an increase in the level of inflammatory indicators, which could lead to elevated metabolic syndrome risk^45^.

A systematic review of the relationship between DII score and lipid profiles revealed that there was a direct association between the dietary inflammatory index score and plasma triglyceride levels in healthy subjects^46^. Although the outcomes of the abovementioned investigations confirmed the correlation between DII score and dyslipidemia incidence, nontraditional lipid indices have not been fully investigated. Therefore, additional studies of various age groups with different genetic backgrounds are necessary to investigate this correlation.

On the other hand, an investigation of individuals with metabolic syndrome revealed that increasing the DII had no significant effect on blood HDL-C levels^47^. Moreover, studies on individuals with type 2 diabetes have shown that adherence to a diet with a higher inflammatory index score has no significant relationship with HDL or triglyceride levels^48,49^. Moreover, a study of women with diabetes did not reveal a significant connection between following a proinflammatory diet and improving LDL-C/HDL-C^50^.

The present study emphasizes the correlation between DII score and atherogenic risk. Research on children has revealed that dietary inflammation, as evaluated by the C-DII, is directly related to atherogenic risk^51,52^. Additionally, studies on Dutch elderly individuals highlighted that atherogenic indices were more desirable in people fed anti-inflammatory diets^39^. Research on healthy adults^45,53^, children and adolescents^54,55^ has also emphasized the direct relationship between DII scores and atherogenic risk. In contrast, a study conducted by Behbahani et al. showed no significant relationship between the dietary DII score and atherogenic risk^21^.

However, further studies are needed to assess the interdependency of DII scores with dyslipidemia and atherogenic risk in individuals with different levels of inflammatory indices as well as different disorders.

In the present study, individuals with the highest DII score (fourth quartile) had significantly greater intake of energy and less intake of selenium, dairy products, and calcium. (Compared to the lowest level, P value ≤ 0.001). Studies have revealed that increased energy intake, especially at the end of the day (simultaneously with a decrease in insulin sensitivity that has a daily rhythm), may lead to a decrease in lipoprotein lipase activity and ultimately could increase plasma LDL cholesterol levels. However, additional studies are needed to investigate the link between energy consumption patterns and the studied complications^56^.

It should be noted that Selenium intake is necessary for physiological processes (in combination with certain proteins called selenoproteins). Hence, consuming an appropriate amount could play an effective role in improving plasma triglyceride and cholesterol levels^57,58^.

Selenium increases 5-deoxy prostaglandin J2A production, which is shown in the legend. It is known for its role as a peroxisome proliferator-activated receptor (PPAR-γ). Research has shown that its activity can reduce cholesterol synthesis by decreasing the sterol regulatory element-binding protein-2 (SREBP-2) concentration^59,60^.

In addition, a low selenium level reduces the expression of HMG-COA reductase (an enzyme necessary for cholesterol synthesis), which subsequently leads to a rise in the concentration of lipids that breakdown in the bloodstream. Additionally, studies have shown a positive correlation between selenium levels in erythrocytes and cardiovascular risk parameters^61–63^.

In addition, it is assumed that there is a direct correlation between dietary calcium intake and lipid profile improvement^64^. The beneficial effects of calcium on ameliorating serum lipids and lipoprotein concentrations can be justified through various mechanisms, including reducing the absorption of fatty acids and increasing their excretion through feces (by forming insoluble calcium-fatty soaps in the gut, the ability to bond with bile acids, and boosting the conversion of cholesterol into bile acids, which leads to increased cholesterol excretion)^65,66^. Moreover, intracellular calcium promotion in hepatocytes could stimulate microsomal triacylglycerol transfer protein, which is involved in VLDL synthesis and excretion from hepatocytes^64^. If dietary calcium intake is limited, calcitrophic hormones increase calcium intake from adipocyte cells, which ultimately leads to a decrease in calcium in adipocytes and a subsequent increase in lipolysis^67^.

According to recent studies, dairy products are rich sources of calcium and other nutrients, including proteins and fatty acids, and there seems to be an inverse relationship between dairy product consumption and the investigated indicators (dyslipidemia and atherogenic risk)^68^. Dairy products contain whey proteins (including whey and casein), which are regarded as crucial factors for improving lipid profiles and reducing CVD risk (through reducing postprandial triglycerides)^69^. Although these products are rich in saturated fatty acids, monounsaturated fatty acids (such as oleic acid) constitute approximately 25% of the fat content in these products and may improve lipid profiles through insulin resistance and limitation pathways^70^. Moreover, the high mineral content in dairy products (calcium, potassium and magnesium) is related to their effectiveness in improving lipid profiles and reducing atherogenic risk^71,72^.

## Strengths and limitations

This study has several noteworthy strengths. First, the sample size was large, providing greater statistical power to detect associations. Second, confounding factors were adjusted for in the analysis, which enhances the validity of the findings. Moreover, this study is the first to investigate the relationship between nontraditional lipid profiles and diet, providing novel insights into the potential impact of dietary habits on lipid metabolism. Despite its strengths, there are some limitations. First, the causal relationships cannot be inferred because of the cross-sectional design of the current study, which means more prospective studies are needed to establish the results conclusively. Second, dietary intake was assessed by food-frequency questionnaire (FFQ), hence we could not deny recall bias and misclassification of study participants. Additionally, owing to the questionnaire setting, only 31 out of 45 parameters were extracted from FFQ. However, a previous study found that there would be no drop-off in the predictive ability of the DII if only 28 parameters were used in predictive ability^73^. As a result, conducting further well-designed longitudinal studies while tackling these limitations is highly recommended.

In summary, the study findings showed that adherence to a diet with a high inflammatory score increases the atherogenic indices.

## Data Availability

The data sets generated during this study are available from the corresponding author upon reasonable request via email.
